# Challenges and patient outcomes in chronic subdural haematoma at the level of a regional care system A multi-centre, mixed-methods study from the East of England

**DOI:** 10.1093/ageing/afae076

**Published:** 2024-04-12

**Authors:** Daniel James Stubbs, Sam Khanna, Benjamin M Davies, Mark E Vivian, Tom Bashford, Krishma Adatia, Ping Chen, Peter John Clarkson, Catherine McGlennan, Lalani Indurawage, Martyn Patel, Rada Tyagunenko, Rowan Burnstein, David K Menon, Peter J Hutchinson, Alexis Joannides, on behalf of the SENIOR-C team

**Affiliations:** Division of Anaesthesia, University of Cambridge, Addenbrooke’s Hospital, Hills Road, Cambridge, CB2 0QQ, UK; Department of Perioperative, Acute, Critical, and Emergency Care (PACE), University of Cambridge, Addenbrooke’s Hospital, Hills Road, Cambridge CB2 0QQ, UK; Department of Clinical Neurosurgery, University of Cambridge, Addenbrooke’s Hospital, Hills Road, Cambridge CB2 0QQ, UK; Department of Anaesthesia, Cambridge University Hospitals NHS Foundation Trust, Addenbrooke’s Hospital, Hills Road, Cambridge CB2 0QQ, UK; Department of Anaesthesia, Cambridge University Hospitals NHS Foundation Trust, Addenbrooke’s Hospital, Hills Road, Cambridge CB2 0QQ, UK; Department of Engineering, Health Systems Design Group, Trumpington Street, Cambridge CB2 1PZ, UK; Department of Anaesthesia, North West Anglia Foundation Trust, Peterborough City Hospital, Peterborough PE3 9GZ, UK; Department of Anaesthesia, Queen Elizabeth Hospital Kings Lynn NHS Foundation Trust, Gayton Road, Kings Lynn, PE30 4ET, UK; Department of Engineering, Health Systems Design Group, Trumpington Street, Cambridge CB2 1PZ, UK; Department of Anaesthesia, Bedfordshire Hospital NHS Foundation Trust, Luton and Dunstable University Hspital, Lewsey Road, Luton, LU4 ODZ, UK; Department of Anaesthesia, James Paget University Hospitals NHS Foundation Trust, Lowestoft Road, Gorleston-on-Sea, Great Yarmouth NR31 6LA, UK; Older People’s Medicine Department, Norfolk and Norwich University Hospitals NHS Foundation Trust, Colney Lane, Norwich NR4 7UY, UK; Clinical Associate Professor in Translational and Clinical Medicine, Norwich Medical School, University of East Anglia, Norwich, UK; Department of Anaesthesia, Northwest Anglia NHS Foundation Trust, Hinchingbrooke Hospital, Parkway Hinchingbrooke, Huntingdon PE29 6NT, UK; Department of Anaesthesia, Cambridge University Hospitals NHS Foundation Trust, Addenbrooke’s Hospital, Hills Road, Cambridge CB2 0QQ, UK; Department of Medicine, University of Cambridge, Addenbrooke’s Hospital, Hills Road, Cambridge CB2 0QQ, UK; Department of Clinical Neurosurgery, University of Cambridge, Addenbrooke’s Hospital, Hills Road, Cambridge CB2 0QQ, UK; Department of Clinical Neurosurgery, University of Cambridge, Addenbrooke’s Hospital, Hills Road, Cambridge CB2 0QQ, UK

**Keywords:** chronic subdural haematoma, perioperative medicine, epidemiology, care systems, older people

## Abstract

**Background:**

Chronic subdural haematoma (cSDH) is a common neurosurgical pathology affecting older patients with other health conditions. A significant proportion (up-to 90%) of referrals for surgery in neurosciences units (NSU) come from secondary care. However, the organisation of this care and the experience of patients repatriated to non-specialist centres are currently unclear.

**Objectives:**

This study aimed to clarify patient outcome in non-specialist centres following NSU discharge for cSDH surgery and to understand key system challenges. The study was set within a representative neurosurgical care system in the east of England.

**Design and methods:**

We performed a retrospective cohort analysis of patients referred for cSDH surgery. Alongside case record review, patient and staff experience were explored using surveys as well as an interactive c-design workshop. Challenges were identified from thematic analysis of survey responses and triangulated by focussed workshop discussions.

**Results:**

Data on 381 patients referred for cSDH surgery from six centres was reviewed. One hundred and fifty-six (41%) patients were repatriated following surgery. Sixty-one (39%) of those repatriated suffered an inpatient complication (new infection, troponin rise or renal injury) following NSU discharge, with 58 requiring institutional discharge or new care. Surveys for staff (*n = 42)* and patients (*n = 209*) identified that resourcing, communication, and inter-hospital distance posed care challenges. This was corroborated through workshop discussions with stakeholders from two institutions.

**Conclusions:**

A significant amount of perioperative care for cSDH is delivered outside of specialist centres. Future improvement initiatives must recognise the system-wide nature of delivery and the challenges such an arrangement presents.

## Graphical Abstract

**Graphical Abstract:**
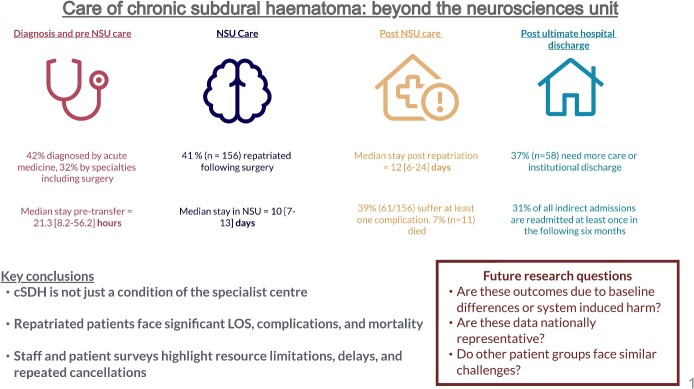

## Key Points

- This study is the first evaluation of outcomes following repatriation after surgery for chronic subdural haematoma.
- Post neuroscience unit discharge patients face: significant hospital stay (median = 12 days), new complications (~40%) and increased dependency (37%)
- Under resourcing, communication difficulties, and inter-hospital distances are key challenges in the regional care of chronic subdural haematoma.

## Introduction

Chronic subdural haematoma (cSDH) is a common neurosurgical condition with a collection of altered blood products forming between the dura and brain. Chronic subdurals appear either *de novo* or on the background of antecedent trauma [1], perhaps mediated by a chronic inflammatory process [2]. At a population level, cSDH is associated with age and is common among cohorts of older emergency neurosurgical patients [3]. Patients often have co-existing comorbidities and are frequently taking pre-morbid antithrombotic medications [4, 5]. Shifting demographics are projected to lead to a 50% rise in operative workload over the next 20–30 years [6, 7].

Surgery for cSDH can yield significant improvement in neurological function [8] and survival [9]. However, retrospective analyses demonstrate a significant rate of perioperative morbidity [5], with the use of electronic health records (EHR) demonstrating that the degree of organ dysfunction within the NSU may be more prevalent than previously thought [4]. Given well documented issues with neurosurgical bed capacity [10] and rising case rates [6, 7], addressing in-hospital morbidity is of crucial importance.

In the United Kingdom (UK) surgical care is concentrated in tertiary centres (neurosurgical units—NSU) [11, 12] with patients referred for advice and intervention. In some regions over 90% of patients who undergo surgery for cSDH are diagnosed elsewhere before being transferred for surgery [4], with 47% of postoperative NSU patients transferred to other hospitals for ongoing care [5].

The siloed nature of care between institutions means that routinely collected data does not cohesively record complications, or care challenges across the patient journey, risking overlooking key problems in care delivery.

In other frail surgical cohorts, multidisciplinary guidelines have led to dramatic improvements in care [13]. Replicating such an approach for patients whose care requires transfer between centres (including cSDH and other tertiary surgical pathologies) requires a detailed understanding of how care is delivered in referring and repatriating centres, who is involved, and the challenges at different stages of the pathway.

In this project, based in a representative neurosurgical referral network in the UK we sought to conduct the first evaluation of care before, and after, NSU stay for patients undergoing surgery for cSDH while simultaneously seeking to better understand the structure and function of the wider system of perioperative care. We used iterative and overlapping techniques, including the evaluation of a retrospective cohort of regional patients, paired staff and patient surveys, system mapping, and a workshop of key stakeholders.

## Methods

### Setting

Cambridge University Hospitals NHS Foundation Trust (CUH) is the regional NSU for most of the East of England (EoE), a population approaching 5 million people. All referrals are recorded in a referral database (Orion).

### Approvals and set-up: Evaluation of outcome and survey

After discussion with the CUH research and development (R&D) team these project phases were deemed a multi-site service evaluation. Registration was made with the CUH quality and safety team (reference PRN8889) and project methods and data sharing approved by the CUH Caldicott guardian and information governance teams. Project set-up was grounded in the recognition that the ‘local’ clinical care team for this patient cohort is, by design, distributed across institutions.

All referring hospitals were approached to participate. Members of relevant clinical specialties in each centre (including acute and emergency medicine, anaesthesia, and geriatric medicine) were approached to act as local evaluating teams. Project details were shared to allow registration in each hospital. Approval was gained from the Caldicott guardian in each referring hospital. No data were shared without bilateral approvals from each institution and completion of a data-sharing agreement.

### Approvals and setup: Workshop

The convened stakeholder workshop was conducted under pre-existing ethical approvals as part of the ‘Designing Improved Surgical Care for Older people’ study, approved by the London and Surrey Borders research ethics committee (19/L0/1648) in September 2019. All workshop participants gave written informed consent to participate.

### Cohort identification and data-sharing

Patients who underwent surgery for cSDH between November 2014 and March 2020 were identified from a referrals database. NHS numbers of patients referred from each centre were securely shared with referrers via secure NHS.net email. No personally identifiable data was shared outside of professionals who could constitute the broader care team. A ‘key’ mapped each NHS number to a unique study ID that was used for all subsequent communication. A standardised data-collection form was used by the collecting team in each hospital and returned by NHS.net email. At no point did any data leave NHS IT infrastructure.

### Variable definitions and data collection

Admitting medical teams and transfer times to the NSU were recorded and key professionals visualised on a stakeholder diagram (Supplementary Figure S1). Following repatriation we collected; re-admission date, accepting specialty, details of medical complications (new antibiotics as a surrogate for infection, rise in troponin, or creatinine rise of more than 50% above baseline), as well as details of ultimate discharge date and destination. Comorbidity and other baseline patient data were derived from admission notes/records in referring centres and reported via data collection template. Data were summarised using descriptive statistics with continuous variables expressed as median [interquartile range].

### Survey development

A free-text staff survey was developed and approved with our quality and safety department. The questionnaire (supplementary material) was designed to address key rhetorical questions used to guide a ‘systems approach’ to healthcare improvement [14]. Questionnaires were distributed electronically (www.qualtrics.com). The survey sought to gain views on structure of the cSDH regional care system, professionals involved in caring for this cohort, and major challenges. Questions seeking to understand system structure and function were used to inform system maps for the workshop.

Patient questionnaires were developed with the CUH patient experience department for appropriateness and clarity. Surveys were distributed by post to all patients alive at the point of distribution who had undergone surgery for cSDH within the study period. Full questionnaire is shown in supplementary material. In this report, to allow pooling of information between staff and patient surveys, free-text responses to four questions were examined (Supplementary Table S1). Key demographic and experience responses to multiple choice questions are reported for context.

### Survey analysis

Free-text responses were analysed using inductive thematic analysis. Each free-text response was coded independently by two authors and arbitrated by a third to define final codes (Supplementary Table S2). Multiple codes could be applied to individual comments. Frequency of codes was then mapped to key stages of the patient journey.

### Stakeholder workshop

We convened a face-to-face workshop of key stakeholders based on survey responses. The workshop aimed to validate findings of results from earlier project phases and consolidate understanding of key challenges. Sampling was purposive, with snowball sampling attempted to access staff groups in other centres. Members of our regional collaborative (see acknowledgements) were approached to take part.

Prior to the workshop, process maps of regional care pathways (see supplemental material) were developed based on group understanding and survey responses. Workshop participants used these to localise difficulties to points on the pathway. Challenges were identified through small group discussion structured around a stereotyped case (a ‘persona’) [15], the content of which was informed by earlier analysis of institutional [4] and national data [5]. Discussions were audio recorded and transcribed using an automated service (Otter.ai) with transcriptions checked for accuracy. Key challenges were identified from review of these transcripts and contemporaneous observer notes kept by two medically trained observers. For clarity, challenges were represented in the form of a causal diagram. Further methodological details are available in the supplemental material.

## Results

### Participating sites, survey responses and cohort characteristics

Six hospitals agreed to participate, covering *n = 494* (55%) of the 900 cSDH referrals received between November 2014 and March 2020. Outcome data were received for 381 (78%) of these. Cohort characteristics and clinical features at index admission are shown in Table 1. A crucial trigger for this study is the observation that approximately 90% of operative cSDH cases are referred from another hospital provider [4].

**Table 1:** Baseline characteristics of a cohort of patients undergoing surgery (*n = 381)* for chronic subdural haematoma, initially referred from a non-neurosurgical hospital. ^*^antithrombotic = any antiplatelet or anticoagulant medication

Characteristic	Median [IQR] or n (%)
Age	78 [70–85]
Male	257 (67)
Admitted from own home	338 (89)
**Mechanism of injury**	
*History of fall*	213 (56)
**Baseline comorbidity**	
*Ischaemic Heart Disease*	92 (24)
*Arrhythmia*	80 (21)
*Prosthetic heart valve*	3 (0.7)
Antithrombotic^*^	155 (41)
*Cerebrovascular disease*	61 (16)
*Dementia*	42 (11)
*Diabetes*	73 (19)
*Chronic Kidney Disease*	42 (11)

Staff surveys were distributed electronically to participants identified by leads in each hospital. In total *n = 42* responses were received. Twenty four responses (57%) were from doctors, 15 (36%) from nurses, two (5%) from physiotherapists and one from an operating department practitioner.

Patient surveys were distributed to *n = 430* patients who had surgery at CUH within the study period and were alive at survey posting (July 2021). In total, 209 (48.6%) partial or complete responses were received.

### Diagnosing specialities, stakeholders, and pre-NSU stay.

Commonest referring specialities prior to transfer were acute, emergency, and general medical specialities (*n = 148, 42%)*. Others included medicine for older people (*n = 67, 19%*), stroke (*n = 26, 7%)*, and ‘other’ (including general surgery and trauma and orthopaedics) (*n = 114, 32%).* Median length of stay (LOS) prior to NSU transfer was 21.3 hours [8.2–56.2] for the *n = 347 (*91%) of patients for whom admission and discharge times were available. Median stay by centre ranged from 12 to 24 hours.

A full list of stakeholders involved in the diagnosis and care of patients with cSDH are summarised in the stakeholder diagram shown in Supplementary Figure S1.

### Repatriation and post-discharge course

**Figure 1:**
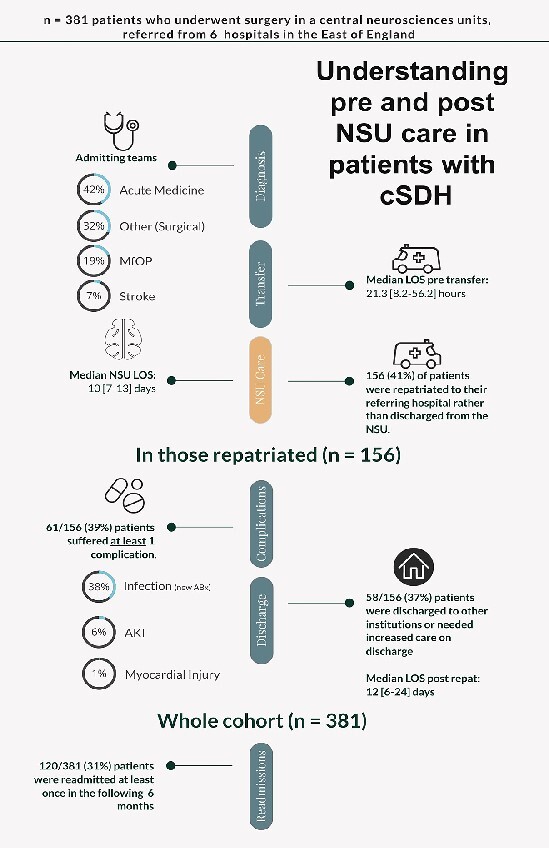
Major findings of pre and post neurosciences unit (NSU) care in patients with a chronic subdural haematoma. The study examines the care and outcomes of 380 patients referred for surgery at a central NSU from six referring centres. Data from the NSU perspective suggests approximately 90% of all operative cases [4] may be referred from another hospital provider prior to surgery. MfOP = Medicine for older people

156 (41%) of patients were repatriated following surgery. Details on re-admitting team were available for 147 (94%) of these. This was most commonly medicine for older people (*n = 67, 46%)* with neurology (*n = 28,* 19%), general medicine (*n = 27,* 19%), stroke (*n = 20*, 13.6%), rehabilitation (*n = 2,* 2%), or other (including surgical specialities) (*n = 3,* 2%) also represented*.* Median NSU LOS for those repatriated was 10 days [7–13]. Rates of repatriation between centres ranged from 26 to 70%. Median LOS following repatriation was 12 days [6–24]. Major findings are summarised in Figure 1.

59 (38%) of those repatriated received new inpatient antibiotics. Nine (6%) exhibited a rise in serum creatinine of 50% or greater from baseline. Two (1%) had a documented troponin rise. Sixty-one (38.3%) repatriated patients suffered at least one complication. Following repatriation 11 (7%) patients died as an inpatient.

Of 156 repatriated patients, 98 (62.8)% were discharged to their own home. Discharge destinations for those initially admitted from their own home (*n =* 135–86.5%) are shown in Table 2. A total of 43% of these patients required onwards discharge to another facility or an increase in home care package.

**Table 2:** Discharge destinations following repatriation in the subset of patients who were initially admitted from their own home (135 of 156)

	N (%)
Own Home	63 (47)
Home with increased care	16 (12)
Residential Home	8 (6)
Nursing Home	9 (6)
Other Hospital Facility	25 (19)
Missing/unavailable	14 (10)

There were 138 re-admissions to the referring centre, in the 6 months following discharge. Ninety (23.6%) patients had at least one readmission, with 30 (8%) having two or more episodes.

### Thematic coding of survey responses

**Figure 2:**
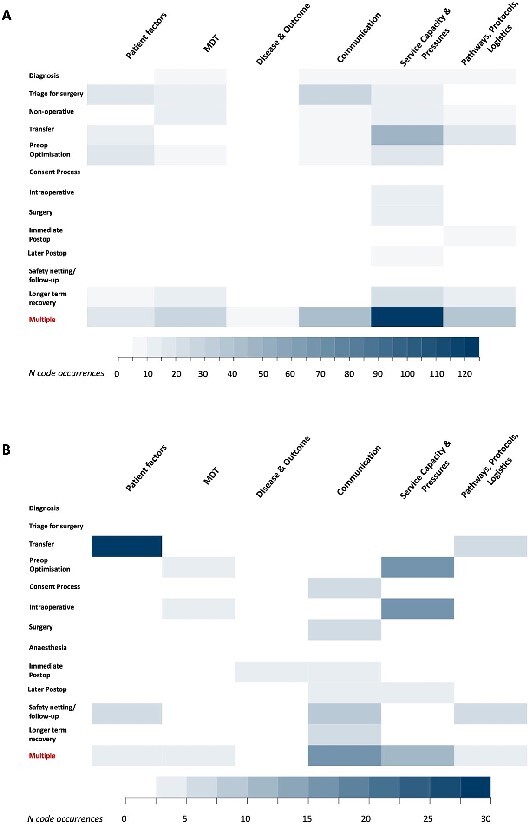
Frequency of challenges in the delivery of regional care for cSDH identified via staff (A) and patient (B) surveys. Figures demonstrate co-localisation of major challenge themes (see supplemental material for details on coding), mapped to phase of care. Colour intensity refers to number of instances of specific codes drawn from 80 free-text comments, from *n = 41* staff and 97 free-text comments from *n = 209* patient surveys. Each passage of text could contain multiple codes.

Analysis of free-text responses to staff and patient surveys (Supplementary Table S2) yielded 76 unique codes across seven major themes (Supplementary Table S2). Each statement was mapped to a major phase of care agreed by the three assessors (Figure 2A and B). These followed the patient journey with minor differences between staff and patient perspectives (e.g. no ‘non-operative’ phase for patient responses as all underwent surgery).

### Staff perspectives on challenges

Eighty free-text responses from 41 respondents across the four included staff questions were reviewed. The most frequent codes were in the ‘Service capacity and pressures’ theme, with 122 occurrences at multiple pathway points. When considering individual phases of care; transfer (*n* = 45) and longer term recovery (*n* = 24) were major issues, with respondents identifying bottle-necks in bed and service availability. Frequency of themes against phases of care are shown in Figure 2A. Communication featured as a major theme; cutting across multiple phases of care *(n = 41)* but especially at surgical triage *(n = 26)*; with difficulty accessing relevant information or neurosurgical advice.

### Summary of patient experience

Of 176 respondents who indicated their age, 74% (*n = 139*) were aged 70 or over. 30% (*n = 62*) of respondents completed the survey with help from a relative or carer. Only 33 (16%) indicated that they were discharged to another hospital at the conclusion of their NSU stay, suggesting relative under-sampling of this group compared to the proportion of admissions.

A total of 181 (87%) individuals gave an overall rating of their care in the NSU using a 10-point scale. Modal response was 10 (*n = 87*). Only eight individuals rated their care as six or lower.

Forty eight (of 132, 36%) respondents indicated that their operation had been cancelled at some stage, with 28 reporting an entire day without eating and drinking due to delays. Most responses to a question discussing ease of receiving family visiting (146/177) identified no major barriers. However, 32 individuals indicated that such visits either couldn’t occur or were difficult.

### Patient complaints

Details on complaints registered with referring centres were also sought by local teams. Four (1%) complaints were registered across three institutions. Complaints related to falls, discharge concerns, and diagnostic delays. Details of NSU complaint data for this cohort are published elsewhere [16]. Key themes for NSU complaints related to cancellation, communication difficulties and falls [16].

### Patient perspectives on challenges

Ninety seven free-text responses were received to four free-text questions. The highest concentration of responses occurred in *‘*Patient factors’ (including considerations such as; normal place of residence, comorbidities, and social support structure) and ‘Transfer’—with difficulties in family visiting a key issue. Communication challenges at multiple stages were also raised (*n* = 16) as well as capacity issues leading to cancellation (*n* = 16 for both).

### Workshop—Attendees

Sixteen individuals, representing 11 major stakeholder groups attended (Supplementary material Table S3). On the day of the workshop, two participants were unable to attend due to coronavirus pressures. Allowing for non-attendees, 11 (69%) participants were male. The patient representative had personal experience of cSDH and their care had required inter-hospital transfer. Four attendees had either current or prior experience in caring for patients with cSDH in referring hospitals. Managerial representative had insight into funding structures covering regional care pathways (neuroscience and stroke).

### Workshop—Localisation of challenges

Participants were asked to highlight major challenges on two representations of the patient journey. One was patient-centric, representing the change in patient ‘state’ as their care progressed [17], the other a process map. Each attendee could leave three labels using a traffic-light system of priority (red = most significant) (Supplementary Figure S2). Of 48 labels, 34 (68%) were placed on the process map. Eleven (23%) were positioned at points representing neurosurgical referral and transfer, with a further five (10%) at non-operative and local management. Six (13%) votes were positioned at the ‘medical optimisation step’, with equivalent numbers relating to ‘awaiting theatre’. Nine (19%) related to postoperative management.

### Workshop—Nature of challenges

**Figure 3:**
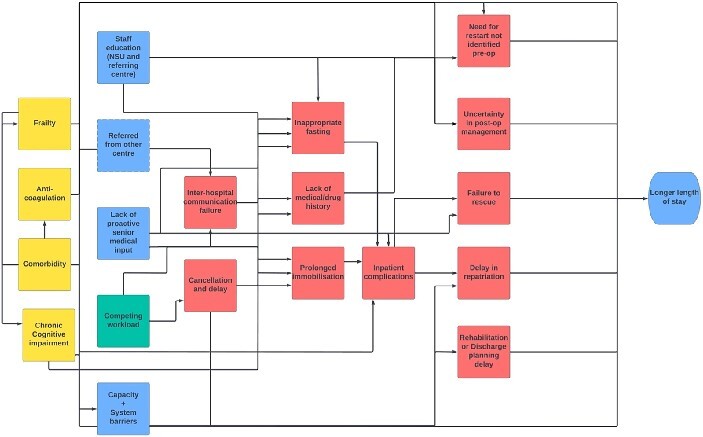
Summary causal diagram demonstrating how key challenges in the care of patients with chronic subdural haematoma may lead to longer length of stay. Diagram developed from review of audio transcripts of stakeholder workshop and field notes of two observers. Edges (arrows) simplified for clarity—full edge index available in supplemental material. Yellow boxes = baseline patient factors, blue boxes = structural system factors (for cSDH), green = external pressures, red = emergent events that could mediate longer length of stay. *Failure to rescue* refers to a system failure in identifying and reacting to emergent perioperative complications.

Putative relationships between challenges were inferred from audio descriptions and expert knowledge. These were expressed in the form of a causal diagram that was subsequently reviewed by three authors, centred around earlier, statistically significant, results from an analysis of our EHR [4]. The resultant diagram suggests that NSU LOS may be impacted by factors across secondary and tertiary care (Figure 3**).**

## Discussion

This is the first study to evaluate care delivery and patient outcome for cSDH at a regional level. As well as evaluating pre and post-operative care pathways we have attempted to triangulate challenges through multiple methods. These mixed approaches, with due reference to earlier qualitative [16] and quantitative [4] work from our centre, allows us to reposition cSDH and its care as a disease of relevance to regional health systems, not just specialist centres. Arguably, similar challenges may face patients with other conditions requiring tertiary surgical input (such as vascular surgery) and so, methodologically, our approach is also of relevance to those involved in the broader perioperative care of such patients. Collating this data posed significant logistical challenge and as such our data collection fields were intentionally simplified, limiting our ability to offer detailed breakdowns. The challenges in data collection, importance of our hypothesis generating findings, and scale of cross-institutional care in these settings means efforts to reliably capture such data at scale is urgently required.

The scale of care provided by referring hospitals is significant. Repatriation rate is high (over 40%) with significant post-surgical LOS in the referring hospital (median = 12 days). This is associated with significant rates of new infection and care needs at the point of ultimate hospital discharge. Concerningly, mortality in the repatriated sub cohort is substantially higher (7% versus 2.4%) than that seen in the NSU [4]. Comparable data on care requirements in those discharged from the NSU is not available from our cohort. However, national data suggests that approximately 8% of all admissions required caregivers when discharged home [5]. Our figure (of 12%) for the number needing a care package is slightly higher but, when viewed in context of the number needing new institutional discharge (Table 2), it suggests that care requirements in those repatriated may be higher than those discharged from the NSU. Whether these figures reflect baseline differences in comorbidity or case complexity, or an emergent consequence of cross-institutional care is unclear and should be a subject of further investigation.

The rate of infection observed in repatriating hospitals (84% of 156 repatriated) is higher than rates of pneumonia or surgical site infection in a nationwide survey of NSU cases (9%) [5]. This could be explained by case ascertainment (with our use of antibiotic prescriptions potentially overly sensitive) or that the cohort of repatriated patients may be more unwell, hence their repatriation rather than discharge from the NSU. We do not have a breakdown on antibiotic indication (and thus infection source), an intentional choice to simplify data collection and minimise issues with cross-institutional differences in antibiotic guidance. National data (from the NSU) suggests that pneumonia is the most commonly observed infection in that setting [5], but this requires corroboration at the level of the referring hospital. Also, this study did not include urinary tract infection in its specified complications [5]. This would be compatible with cSDH leading to impaired consciousness and prolonged immobility, especially following surgery but understanding the breakdown of infections by site (e.g. urinary, chest, surgical site) and precipitating causes requires further prospective work. We are currently planning such studies at a national level to better understand how NSU care impacts on post-repatriation health trajectory and whether similar challenges may face other cohorts requiring transfer prior to expedited (but not time-critical) specialist surgery.

As well as attempting to describe care structure across both secondary and tertiary care, we also attempted to understand staff and patient challenges through paired surveys, as well as a stakeholder workshop. Overall, patients were very satisfied with their care. However, survey responses were heavily skewed towards those discharged directly to their own home from the NSU. This, coupled with the outcome data presented in this study and the duration of time covered (2014–2020) means it is possible our responses disproportionately reflect the experience of those with a favourable outcome. If this were the case, we would hypothesise that our survey data reflects an over-optimistic view of patient experience. Further prospective studies or alternative approaches (such as interviews or ethnography) may offer additional strategies to better understand the breadth of patient perspectives.

Although overall satisfaction was high, the commonest challenges patients identified were those of distance, with communication and family visits impacted by resultant travel difficulties, and delay (Figure 2B**).** Importantly, surgery in all cases occurred before the SARS-CoV2 pandemic. Pre and post-operative transfer was identified as a ‘bottle-neck’ by staff respondents who highlighted delays waiting for specialist beds and surgical triage. Workshop discussions confirmed these findings (Supplementary Figure S3 and Figure 3) with pathway resourcing, communication, and provider insight highlighted as potential drivers (see quotations in supplemental materials). Workshop participants suggested that the relative rarity of the condition (from the perspective of a clinician in a non-specialist centre) may reduce familiarity and create an educational need, a finding consistent with gaps in staff and patient educational resources [18].

Our study has several limitations. Firstly, data collection for the project ran through the second and third quarters of 2021. The SARS-CoV2 pandemic limited our ability to corroborate survey findings with individual hospital visits (as originally planned) and could have adversely affected both survey response and data collection from referring sites. We recognise that our survey responses are likely skewed, with staff responses especially low, considering the number of centres involved. However, the triangulation of these findings from our workshop data helps support the relevance of these results. Although our results reflect care delivered across seven institutions, we acknowledge that this remains an evaluation of care across a single neurosciences service and, as such, the generalisability of our findings is uncertain. However, the major themes of our work resonate with the output of a national working group with representatives from multiple UK institutions [19].

## Conclusions

This study describes the regional distribution of care and care challenges for patients undergoing CSDH surgery in one UK referral network. Nearly 40% of patients are repatriated to secondary care following surgery, where they exhibit a prolonged stay, and significant care needs at the point of discharge. The distributed care system for these patients may impair communication and impose logistical and service constraints at multiple pathway points. Understanding the impact of system structure on patient care and experience for those requiring transfer prior to cSDH surgery is a clear priority and could be of relevance to other cohorts requiring expedited, specialist, surgery.

## Supplementary Material

aa-23-1846-File006_afae076
