# Supplementary material for: Challenges and patient outcomes in chronic subdural haematoma at the level of a regional care system A multi-centre, mixed-methods study from the East of England
Source: Age Ageing. 2024 Apr 12;53(4):afae076. doi: 10.1093/ageing/afae076 (PMC11014781; doi:10.1093/ageing/afae076)
Supplement: aa-23-1846-File006_afae076 [file aa-23-1846-file006_afae076.pdf]

Supplementary Material for:

Challenges and patient outcomes in chronic subdural haematoma at the level of a regional care system. A multi-centre, mixed-methods study from the East of England.

Contents:

1. **Key questions from staff and patient surveys used in thematic analysis (Supplemental table S1)**
2. **Staff survey**
3. **Patient survey**
4. **Stakeholder diagram: key roles in the care of cSDH (Supplemental figure S1)**
5. **Thematic coding structure (Supplemental table S2)**
6. Workshop
    a. **Participants (Supplemental table S3)**
    b. **Persona (Supplemental Figure S2)**
    c. **Supplementary methods**
    d. **Localisation of challenges (Supplemental Figure S3)**
    e. **'Morphological charts' used to capture problems and solutions (Supplemental Figure S3)**
    f. **Representative quotations for key problems and summary of challenges**
    g. **Edge index for causal diagram**

Table S1: Key questions from staff and patient questionnaires used for thematic analysis as well as number of individual responses

**Staff survey questions**	***N responses***	**Patient survey questions**	***N responses***
**What factors affect how these patients are looked after in your hospital? This could be events on a large scale (e.g. winter), or a smaller scale (e.g. language barriers)**	**22**	**Please explain why you feel you did not receive enough information**	**29**
		**Please share your thoughts if you feel we can improve the operation experience**	**32**
**What do you need to be able to look after these patients?**	**21**	**If your family was not able to visit you, or it was difficult, please explain why**	**23**
**Are there any things (e.g. equipment, protocols etc…) that are specific or particularly important to the care of these patients?**	**20**	**If you feel information was missing at discharge or information you later realised during your recovery you needed, please provide details what was missing so we can improve discharge information:**	**13**
**What are the current major problems in providing good care to these patients?**	**17**		

Reproduction of staff survey

Staff views on the care of patients with chronic subdural haematoma

We are interested in how patients with chronic subdural haematoma are looked after ***at your hospital, who*** *is involved, and* ***where*** *this care happens. We would be grateful if you could fill in the questions below based on your experiences in looking after these patients. Feel free to use bullet points, notes, sketches, and diagrams to answer the questions.*

Q1 Who is involved in looking after these patients in your hospital?

Please tick all you think are relevant and give us your suggestions of any other staff group!
This could be people who actively meet the patient or are consulted about them or their management…

Emergency Department Doctors		Ward Nurses		Speech and Language Therapists	
Emergency Department Nurses		Specialist Nurses (e.g. outreach)		Discharge planning	
(Acute) Medical Doctors		Physiotherapists		**Any-one else?**	
Anaesthetists		Occupational therapists		·	
Stroke Doctors		Pharmacists		·	
Surgical Doctors		Dieticians		·	

Q2 Where are these patients are looked after in your hospital?

This includes wards or important locations that are involved in the patient journey (facilities, investigations etc…)

Emergency Department		Surgical wards		**Anywhere else?**<br>.<br>.<br>.<br>.
Acute Medical Wards		Radiology		
Stroke Ward		Medicine for the elderly ward		
Intensive Care (or Higher dependency)		Operating theatre complex (incl. Recovery)		
Rehabilitation Ward		Physiotherapy facility		
Neurology ward		Occupational therapy facility		

Q3 What factors affect how these patients are looked after in your hospital?

This could be events on a large scale (e.g. winter), or a smaller scale (e.g. language barriers)

1. ……………………………………………………………………………………………

2. ……………………………………………………………………………………………
3. ……………………………………………………………………………………………

Q4 Which people or organisations might care about how we look after these patients?

They may or may not be directly involved in looking after these patients but would have an interest in how they were looked after or what happens to them.

General Practitioners		Care providers		**y-one else?**<br>.<br>.<br>.<br>.
Other hospital trusts		Charities		
Ambulance Service		Family and Friends		
Clinical Commissioning Groups		Local Authorities		

Q5 Are there any things (e.g. equipment, protocols etc…) that are specific or particularly important to the care of these patients?

1. ………………………………………………………………………………………
2. ………………………………………………………………………………………
3. ………………………………………………………………………………………

Q6 What do you need to be able to look after these patients?

1. ………………………………………………………………………………………
2. ………………………………………………………………………………………
3. ………………………………………………………………………………………

Q7 What are the current major problems in providing good care to these patients?

1. ……………………………………………………………………………………………

2. ……………………………………………………………………………………………

3. ……………………………………………………………………………………………

Q8 Could you outline how you think all of these things (people, places, processes, equipment) work together to look after these patients?

It might be useful to think of where they occur in the journey of a patient with a chronic subdural haematoma

Feel free to concentrate on the parts that you know best (e.g. theatre, ward, discharge) and to continue on another sheet if needed!
Drawings, notes, bullet points, and sketches are all fine!

Reproduction of patient experience survey

The following survey was developed in conjunction with the patient experience team at CUH. Key answers to multiple choice questions are referenced in the text, questions used for thematic analysis highlighted in **Table S1** above. Full results of other questions are not included due to their lack of direct relevance to the messages of this paper.

Question 1: Please tell us who is filling in this questionnaire:

- Patient
- Patient + family member
- Patient + other (caregiver/friend)

Question 2: When did you have your chronic subdural haematoma surgery at Addenbrooke's Hospital?

Question 3: Were you admitted directly to Addenbrooke's Hospital or to another hospital and then transferred?

- Admitted to Addenbrooke's Hospital
- Admitted to another hospital

Question 3b: How long did you have to wait before being transferred to Addenbrooke's?

- Less than a day
- 1 full day
- 2 or more days
- Don't know/Can't remember
- Total

Question 3c: When you arrived at Addenbrooke's, were you admitted to the Emergency Department ('A and E') or were you admitted directly to a ward?

- Emergency Department
- Directly to a ward
- Don't know/Can't remember
- Total

Question 4: Please tick all medications you were taking at the time you were admitted to Addenbrooke's Hospital

- Aspirin

- Clopidogrel
- Ticagrelor
- Warfarin
- Rivaroxban
- Apixaban
- Dabigatran
- Heparin/Dalteparin/Tinzaparin
- Don't know/Can't remember
- Total

Question 5: Before your operation, do you feel you received enough information to prepare you for surgery?

- Yes, completely
- To some extent
- No
- Don’t know/Can’t remember
- Total

Question 6: Before your operation, were you told how you could expect to feel afterwards?

- Yes, completely
- To some extent
- No
- Don’t know/Can’t remember
- Total

Question 7: Before your operation, did a member of staff answer your questions about your operation in a way you could understand?

- Yes, completely
- To some extent
- No
- I did not have any questions
- I was not given the opportunity to ask questions
- I was not able to ask questions i.e. incapacitated to ask questions
- Don’t know/Can’t remember
- Total

Question 8: Do you remember if your operation was cancelled or re-arranged at any stage? If so, were you told not to eat and drink anything for a period of time without actually receiving your operation?

- Yes
- No
- Don't know/Can't remember
- N/A
- Total

Question 9: If your operation was cancelled, how many meals do you think you missed without receiving your operation?

- 1 meal
- 2 meals
- 3 meals – all day with no food
- Don't know/Can't remember
- N/A
- Total

Question 10: After your operation, did a member of staff explain how your operation had gone in a way you could understand?

- Yes, completely
- To some extent
- No
- Don't know/Can't remember
- Total

Question 12: When you had important questions to ask a doctor, did you get answers that you could understand?

- Yes, always
- Sometimes
- No
- I had no need to ask questions
- Total

Question 13: When you had important questions to ask a nurse, did you get answers that you could understand?

- Yes, always
- Sometimes
- No
- I had no need to ask questions
- Total

Question 14: Did you have confidence and trust in the doctors treating you?

- Yes, always
- Sometimes
- No
- Don't know/Can't remember
- Total

Question 15: Did you have confidence and trust in the nurses treating you?

- Yes, always
- Sometimes
- No
- Don’t know/Can’t remember
- Total

Question 16: Did you have confidence and trust in other clinical staff treating you (e.g. physiotherapist, pharmacists?)

- Yes, always
- Sometimes
- No
- I was not seen by any other clinical staff
- Don’t know/Can’t remember
- Total

Question 17: In your opinion, did the members of staff caring for you work well together?

- Yes, always
- Sometimes
- No
- Don’t know/Can’t remember
- Total

Question 18: Were you involved as much as you wanted to be in decisions about your care and treatment?

- Yes, definitely
- To some extent
- No
- Don't know/Can't remember
- Total

Question 19: Did you feel that the care you received was adapted to your own situation?

Yes, definitely
To some extent
No
Don't know/Can't remember
Total

Question 20: Did you feel that any long-term health conditions (such as heart disease or diabetes) were appropriately looked after whilst you were in Addenbrooke's?

- Yes, definitely
- To some extent
- No
- Don't know/Can't remember
- N/A
- Total

Question 21: Did you feel that any long-term medications were appropriately looked after whilst you were in Addenbrooke's?

- Yes, definitely
- To some extent
- No
- Don't know/Can't remember
- N/A
- Total

Question 22: How much information about your condition was given to you?

- Right amount
- Too much
- Not enough
- I was not given any information
- Don't know/Can't remember

Total 183 100%

Question 23: How much information about your treatment was given to you?

- Right amount
- Too much
- Not enough
- I was not given any information
- Don’t know/Can’t remember
- Total

Question 24: Were your family able to visit you whilst you were in hospital?

- Yes, easily
- Yes but it was difficult
- No
- N/A
- Total

Question 25: Please rate your overall care during your stay in Addenbrooke’s Hospital, with 1 being a very poor experience and 10 a very good experience:

- 1
- 2
- 3
- 4
- 5
- 6
- 7
- 8
- 9
- 10
- Total

Question 26: Where were you discharged to when leaving Addenbrooke’s Hospital?

- My own home
- To live with family
- Residential home
- Nursing home
- Another hospital
- Total

Question 26a: If you were discharged to another hospital, where did you go after leaving there?

- My own home
- To live with family
- Residential home
- Nursing home
- Another hospital
- N/A
- Total

Question 27: When you left Addenbrooke's Hospital, did the doctors or nurses tell you what would happen with your care?

- Yes, definitely
- To some extent
- No
- Don't know/Can't remember
- Total

Question 28: When you left Addenbrooke's Hospital did the doctors or nurses give your family, friends or carers all the information they needed to help care for you?

- Yes, definitely
- To some extent
- No
- Don't know/Can't remember
- Total

Question 29: Did Addenbrooke's staff tell you who to contact if you were worried about your condition or treatment after you left hospital?

- Yes
- No
- Don't know/Can't remember
- Total

Question 30: Were you seen in a follow up clinic at Addenbrooke's Hospital?

- Yes
- No, it was offered but appointment not needed
- No, it was not offered and I didn't need an appointment

- No it was not offered and I would have liked an appointment
- Total

Question 31: Please tell us if any of the following could have helped you in your recovery. Please tick all that apply.

- Walking frame
- Walking stick
- Printed chronic subdural haematoma surgery literature
- Support groups
- Contact numbers
- List of online resources
- Total

Stakeholder diagram (Supplemental figure S1)

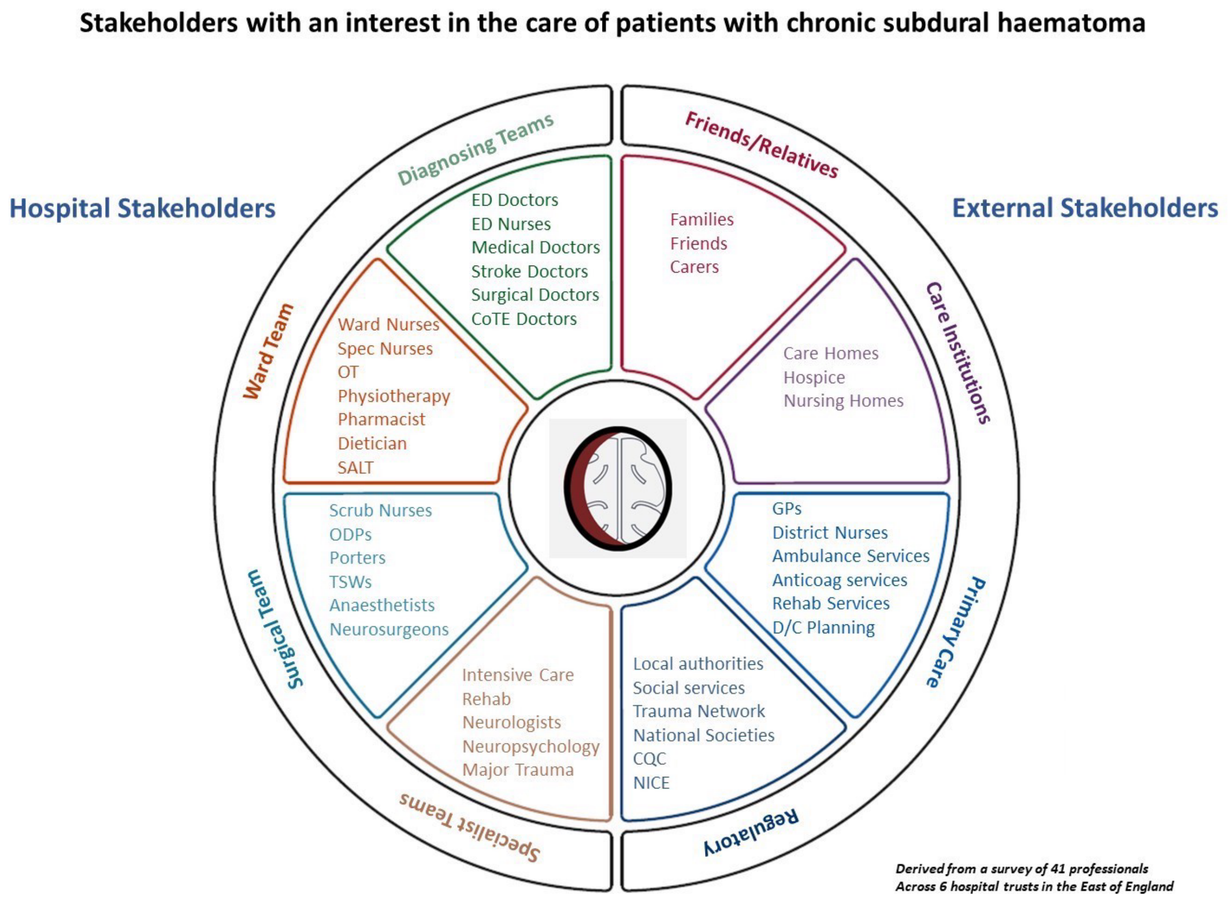

Key stakeholders involved in the care of patients with a chronic subdural haematoma. Template taken from the ‘Improving Improvement’ toolkit (www.iitoolkit.com) - used with permission.

Thematic coding structure (Supplemental table S2)

Organisation of final codes ('Individual code') into major groupings used to map key challenges to phases of care **(Figures 2A and B in main paper)**. All final codes were arrived at following blinded assessment by two senior clinicians and arbitration by a third. Derived from 177 passages of free text (97 from patients, 80 from staff) across eight questions **(Supplemental table S1)** from staff and patient questionnaires.

For anonymity reasons free-text passages of text have been summarised/paraphrased or redacted, preserving major themes. Given anonymous nature of survey sampling the seeking of individualised consent for publishing of direct quotations is not possible.

- Text in square parentheses [ ] = drawn from patient experience survey
- Text in curly parentheses { } = drawn from staff survey

AAGA = Accidental awareness under general anaesthesia, ED = Emergency Department, EEG = Electroencephalography, MDT = Multidisciplinary team (involved in care), SDH related = Subdural haematoma related (specific symptoms or outcomes attributed to the SDH process e.g. weakness, confusion)

MAJOR GROUPING	INDIVIDUAL CODE	CONTENT SUMMARY
**Communication**	Carer	[Family made complaint based on hospitals not communicating need for carer due to memory loss]
**Communication**	Collateral history	{Is important in the care of these patients}<br><br>{(Need to know) Performance status pre surgery}
**Communication**	Communication	[distress due to post-operative complication not aware may occur]

**Communication**	Complaints	[See Carer above]
**Communication**	Consent	[Unaware of specific complications/post-op events]
**Communication**	Holism	{Neurosurgical team require information from communicating teams to make holistic decisions}<br><br>{Effective MDT communication to prevent long stay in hospital}<br><br>{Geriatricians required to give holistic view on ongoing care]
**Communication**	Human factors	{(Variation in) individual decisions from neurosurgeons}<br><br>[Patients report not being aware who their surgeon was, communicating indirectly with them via relatives]
**Communication**	Language	[lack of on-site translator]
**Communication**	Patient concerns	[Concern around drain]<br>[Concern around cancellations]
**Communication**	Patient Diary	[A diary of what happened would have been useful for [RELATIVE] later]
**Communication**	Patient/relative experience	[Family not notified upon completion of operation]<br>[Positive feedback - staff 'always' asking what they could do to help]
**Disease & Outcome related**	?AAGA or Anaesthetic type (LA v GA)	[One report of awareness of staples in wound - unclear if under LA or GA]
**Disease & Outcome related**	Complications	Post-op seizure<br>[Loss of taste and smell - only told related at follow-up]<br>[" very real" hallucinations]

		{Complications impact on how patients are looked after in hospital}<br>{Staff need an awareness of potential complications}<br>Inadequate staff numbers → lack of assessment → deterioration}
**Disease & Outcome related**	Confusion	[Relative confused]<br><br>[Patients report not being aware of things until post-op]
**Disease & Outcome related**	Drain	[Patient concerns re: drain]<br><br>[require] ability of staff to special confused patients (concern due to e.g. drain dislodgement)
**Disease & Outcome related**	Outcome	{Rehabilitation provision is lacking }<br>[Complications (e.g. seizure)]
**Disease & Outcome related**	SDH Related (e.g. natural history)	[SDH] wasn't picked up until [SEVERAL MONTHS AFTER INJURY]
**MDT**	Care of the Elderly	{Those under the care of ortho are often reviewed by care of the elderly consultants but the 'ortho geri' service is under-resourced}<br><br>{[Care of the elderly] input is needed}
**MDT**	ED	Backlog of patients - ED, Radiology, theatres … {makes it challenging to look after these patients}
**MDT**	Intensive Care	[Lack of care on ward following ICU step down]
**MDT**	Neurology	{Lack of a neurology ward [is a current problem]}

**MDT**	Neurosurgery	[Unsure who their operating surgeon was].<br><br>{Access to neurosurgery is important and difficult}
**MDT**	Nursing	{Neurosurgical training for nursing staff (is important)}<br><br>{Skilled nursing (important)}
**MDT**	Nutrition	[long NBM time]<br><br>[Without food all day due to multiple cancellations]
**MDT**	Psychology	
**MDT**	Radiology	Backlog of patients - ED, Radiology, theatres … {makes it challenging to look after these patients}<br><br>Access to radiology {is important}<br><br>Neuroradiology expertise {is important}
**MDT**	Rehabilitation	{Under-resourced}<br><br>{Need for more community rehab}
**MDT**	Stroke	{Patients sometimes admitted to stroke ward}
**MDT**	Surgical Teams	Polytrauma patients may come under surgical teams
**MDT**	Therapy	Adequate therapy staffing - physio, OT, SLT as well as rehab environment [are needed]
**Pathways, Protocols, Logistics**	Discharge	[taxi for discharge]
**Pathways, Protocols, Logistics**	EEG	Access to EEG monitoring {is needed}

**Pathways, Protocols, Logistics**	Equipment	{Communication aids, mobility aids, frames/hoists, pressure relieving mattresses}<br><br>{Monitoring equipment, analgesia, oxygen}
**Pathways, Protocols, Logistics**	Hospital	{Location of beds<br>Specialist v Non-specialist hospitals}
**Pathways, Protocols, Logistics**	Organisational	{Ineffective monitoring devices}<br>[Discharge pathways (e.g. home via taxi)<br>Visiting hours]
**Pathways, Protocols, Logistics**	Other Logistics	{Transfer speed<br>Repatriation logistics}
**Pathways, Protocols, Logistics**	Out of Hours	{Capacity → out of hours operating<br>Agreement between teams on suitability for out of hour operating}<br>[Patients report being fed late at night following cancellations]
**Pathways, Protocols, Logistics**	Pathway	Need for local policies
**Pathways, Protocols, Logistics**	Pressure mattresses	See equipment section
**Pathways, Protocols, Logistics**	Protocols/guidelines	Local protocols for management {are needed}<br><br>{Policies on CT scanning}<br><br>Transfer brain injury guideline/protocol<br><br>Protocols for managing anticoagulation {helpful}<br>Contact details for advice

**Pathways, Protocols, Logistics**	Repatriation	{Need for agreed pathways}
**Pathways, Protocols, Logistics**	Transport/Parking	[Distances make visiting challenging for those without private transport]<br>[Discontinuation of bus routes between home and hospital]
**Pathways, Protocols, Logistics**	Ward	[Insufficient care levels on ward following ICU discharge]
**Patient factors**	Anticoagulation	Anticoagulation management…e.g. When to restart. Need good communication between trusts {problem for this patient group}
**Patient factors**	distance	[See Transport/parking for examples]<br>[Specific distances between geographic locations]
**Patient factors**	Medication	Optimisation of medicines {a problem}<br>Medicines reconciliation {a problem}
**Patient factors**	Patient capacity	{Availability of specialist and non-specialist beds}
**Patient factors**	Patient cohort/needs	[Lack of transport]<br>[Lives alone]<br>[Preop malnutrition]<br>[Preceding trauma]<br>[Poor understanding/recollection of preoperative discussions]
**Patient factors**	Transferred Patients	[Told that 'repatriating hospital' will look after relative]
**Service Capacity & Pressures**	Bed availability	[Both specialist and non-specialist]
**Service Capacity & Pressures**	Competing internal pressures	{Other emergencies<br>General capacity}
**Service Capacity & Pressures**	Delays	{To diagnosis<br>To transfer

		To surgery - competing pressures<br>To repatriation}
**Service Capacity & Pressures**	External pressures (e.g. season, respiratory viruses)	Winter pressures<br>Covid surge
**Service Capacity & Pressures**	Safe	[Reports feeling safe throughout]
**Service Capacity & Pressures**	Safety	[Aggressive/confused patients]<br>[Anticoagulation]
**Service Capacity & Pressures**	Specialist Capacity	Neurosurgery<br>Rehabilitation<br>Care of the Elderly
**Service Capacity & Pressures**	Staff availability	[wish for more '1 to 1' care]
**Service Capacity & Pressures**	Staff skillset/knowledge	Training

Workshop

Participants (Supplemental table S3)

Stakeholder group	Approached	Attended
Consultant Neurosurgeon	3	3
Consultant Anaesthetist	2	1
Consultant Stroke Medicine	1	1
Consultant Geriatrician	2	1
Consultant EM	2	2
Non-consultant Neurosurgeon	3	2
Non-consultant Anaesthetist	2	2
Nurse Specialist	1	1
Operations manager	1	1
Patient	1	1
Physiotherapist	1	1

Persona (Supplemental Figure S2)

Content of the persona was derived from earlier analysis of electronic health record (Stubbs et al. 2020) and complaints (Jones et al. 2021). As well as drawing on findings from national audit data and early outputs of a national working group(Stubbs et al. 2022).

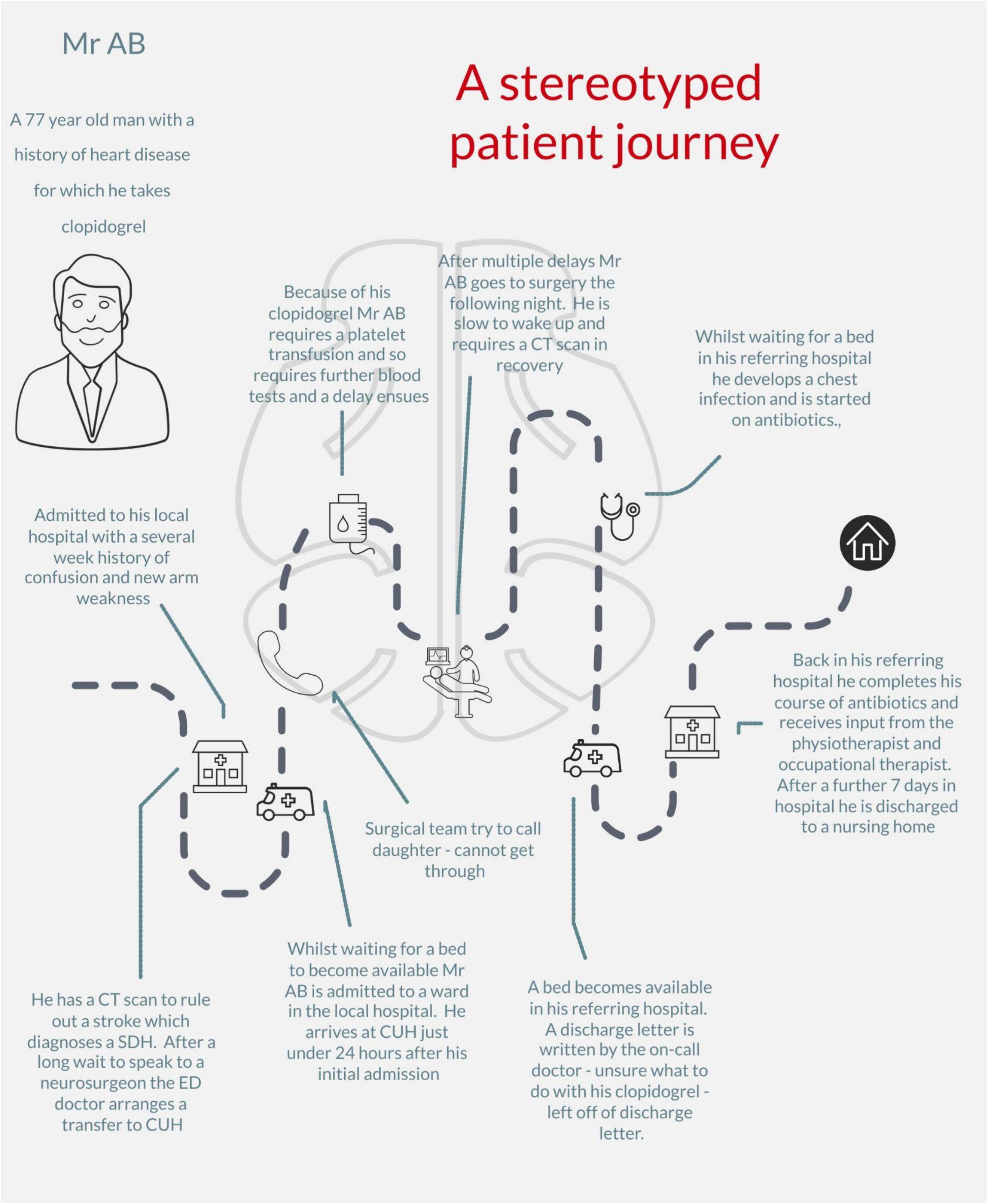

Supplementary methods

Group structures and audio recording

Workshop participants were split into two groups, one examining pre-operative care (including neurosurgical referral and transfer), the second post-operative care and optimisation. One observer was assigned to each group. Facilitators used a persona **(see Supplemental Figure S1)** to guide discussions. Observers documented key problems (and potential solutions) using a structure analogous to a morphological chart [21] (**example given in Supplemental Figure S2**)**.** Challenges from these independently completed charts were reviewed and supporting statements identified from audio transcriptions by an independent party (**see supplemental material**).

Example ‘morphological chart’ to capture problems and solutions (Supplemental Figure S3)

**Problems**	***Idea 1***	***Idea 2***	***Idea 3***	***Idea 4***	***Idea 5***

(text from charts given to both workshop groups is included in the ‘Summary of challenges’ section below.)

Localisation of challenges (Supplemental Figure S3)

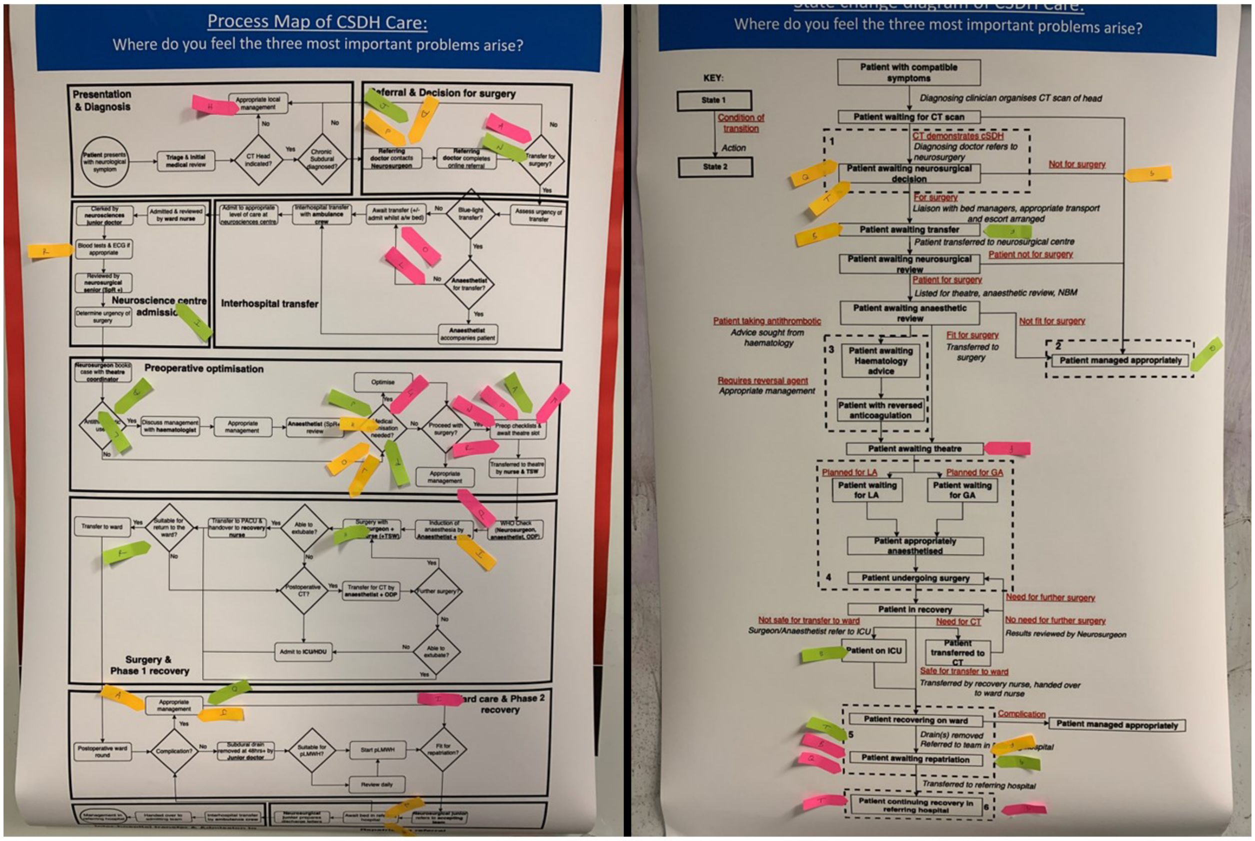

A: Process map of regional care of chronic subdural haematoma patients

B: State change diagram of regional care of chronic subdural haematoma patients

Location of stakeholder identified challenges in patient pathway shown on labelled diagrams from our stakeholder workshop. Participants were asked to label on either diagram where key challenges they encountered in caring for these patients occurred. *Label colour relates to priority in a 'traffic light' system (red = most important)*

Summary challenges and representative quotations

Challenges are drawn from morphological charts (see example in Supplemental figure S2) logged by two independent observers during workshop. Representative quotations mapped from audio transcripts (consent for anonymous use of quotations gained prior to workshop).

**Identifier (and source)**	**Quotation**
**A (Consultant in Emergency medicine from a non- neurosciences unit)**	*"so our problems...issues about professional education, that this is a relatively rare presentation, and one that we may not get that much education about. And then some issues about ... prompt decision making around non operative management, operative management, and the delays in making that decision. And you can't decide which pathway the patient will be going, be it neurosurgical, admission locally, admission to medics, admission to surgeons, until that decision has been made. And then some open issues, particularly at the regional centres, about managing anticoagulation"*
**B: Consultant anaesthetist**	*"We did an audit on the starvation times... And the findings were terrible...some patients were stuck for 72 hours before they went for surgery. They had absolutely nothing to eat and drink...There's no point starving them in the referring hospital. It takes more than six hours for them to come"*
**C: Consultant Neurosurgeon**	*"The surgeons are petrified that if a slot comes up at short notice the anaesthetist will say 'no' [if they've been given something to eat or drink]"*
**D: Consultant Neurosurgeon**	*"it's a dynamic process, you know, today the patient is like 'that', tomorrow, they develop a little bit of chest sepsis or*

	whatever and then they become confused, and everything is very different the next day"
**E: Consultant geriatrician**	*“frailty encompasses, the medical problems, the physical problems, the psychological problems, and potentially some of the deconditioning in terms of that wait from the diagnosis of the subdural to the point where you can actually anaesthetise them”*
**F: Specialist nurse [Neurosurgery]**	*“sometimes you don’t actually know why they’re on the anticoagulant”*
**G: Consultant Neurosurgeon**	*“I think there’s this perception. I think the risk is more whether you have a recurrent risk of falls that could lead to a further haematoma. Actually in neurosurgery, there is no major risk to starting anticoagulation...you know, when you do much more significant surgery, you don’t worry after a few days.”*
**H: Specialist Nurse**	*“So it’s not up to physios, if they walked in, a nurse can get them out of the bed, ... one of my biggest bugbears as a specialist nurse, and when I was senior on the wards, was people writing ‘awaiting physio’, why?”*
**I: Junior doctor**	*“… your point about cross centre communication, I think what comes across is quite variable. Okay. Some centres will send, you know, all the inpatient notes, various ECG, blood tests and some just won't “*

**J Former DGH Medical Registrar**	*… out in the DGH's the general awareness and education of what one does a subdural, I would say it's very low and I would consider myself part of that*
**K Consultant Neurosurgeon**	*Yeah, there's an underlying thing for a lot of things. And that's resource availability.*

The following core concepts (and their putative relationships on patient length of stay) are summarised in **figure 3** in the main paper.

**Core Concept**	**Wording from Morphological Chart**	**Quotation and other sources of evidence**
**Frailty**	Frailty – medical, psychological, deconditioning<br>As per hip fractures<br>What can be optimized and can't be? What is the risk stratification and what can be improved<br>Eg out of hours<br>Nb. cSDH Surgery less problematic/impactful than arthroplasty **(1)**	D<br>E
**Capacity**	Often patient will not have capacity to be involved in the decision making **(1)**	D
**Cancellation and delay**	Out of hours operating **(1)**<br><br>Prompt treatment decision making…very different dependent on neurosurgery…much more ambiguous than NOF [Neck of Femur fracture patients] **(2)**<br><br>No clear pathway for non-operative **(2)**	A<br>B<br>C<br>D<br>K
**Poor inter-hospital communication**<br><br>**Lack of drug/medical history**	Cross-centre decision making and involvement of different teams across different hospitals<br>Care team often remote from patient/family **(1)**<br><br>Basic investigations not necessarily forthcoming. Basic management not necessarily done by source hospital (e.g. bloods) (1)	A<br>B<br>I<br>J

	Variability in terms of documentation patients arrive with **(1)**	
**Competing pressures**	Increase in referrals from 30/day to 80/day in 12 year period (no increase in resources) **(1)**<br><br>Resources **(1)**	Incidence rising see: (Stubbs et al. 2021)<br>K
**Anticoagulation (incl Uncertainty in management)**	uncertainty for why patients are taking these and the need for continuing / restarting **(1)**<br><br>Variability of surgeon response [preference] **(1)**<br><br>Uncertainty about coagulation post neurosurgery **(2)**	A<br>F<br>G<br>J
**Uncertainty in management**<br><br>**Relative inexperience [of staff]**	Professional education (including who takes responsibility of informing the patient of the pathway) **(2)**	A<br>B<br>C<br>F<br>G<br>J
**Comorbidity**		D<br>National audit data (Brennan et al. 2016)
**Repatriation (incl delays)**<br><br>**Discharge planning**<br><br>**Rehabilitation (incl delays)**	Repatriation is a systemic problem **(2)**<br><br>Rehabilitation capacity **(2)**<br><br>Discharge patients (cannot reserve beds…cannot start a care package at [NSU] if going back to local trust concurrently **(2)**	H<br>K
**Cognitive Impairment**<br><br>**[Pre-operative] Anticoagulation**<br><br>**Referred from other centre**<br><br>**Inpatient complications**	Derived from previous analysis of EHR (Stubbs et al. 2020)	A<br>D

Edge index for causal diagram

From	To
Comorbidity	Anticoagulation
Frailty	Chronic cognitive impairment
Inappropriate fasting	Inpatient complications
Prolonged immobilisation	Inpatient complications
Lack of proactive senior medical input	Inpatient complications
Comorbidity	Inpatient complications
Chronic cognitive impairment	Longer length of stay
Need for restart not identified pre op	Longer length of stay
Uncertainty in post op management	Longer length of stay
Frailty	Longer length of stay
Cancellation and delay	Longer length of stay
Failure to rescue	Longer length of stay
Delay in repatriation	Longer length of stay
Rehabilitation or discharge planning delay	Longer length of stay
Referred from other centre	Inter-hospital communication failure
Competing workload	Inter-hospital communication failure
Chronic cognitive impairment	Lack of medical drug history
Inter-hospital communication failure	Lack of medical drug history
Anticoagulation	Need for restart not identified preop
Lack of medical drug history	Need for restart not identified preop
Anticoagulation	Uncertainty in postop management
Staff education (NSU and referring centre)	Uncertainty in postop management
Inter-hospital communication failure	Inappropriate fasting
Cancellation and delay	Inappropriate fasting
Lack of proactive senior medical input	Inappropriate fasting
Staff education (NSU and referring centre)	Inappropriate fasting
Comorbidity	Frailty
Competing workload	Cancellation and delay
Cancellation and delay	Prolonged immobilisation
Lack of proactive senior medical input	Prolonged immobilisation
Staff education (NSU and referring centre)	Prolonged immobilisation
Inpatient complicaitons	Failure to rescue
Lack of proactive senior medical input	Failure to rescue
Inpatient complicaitons	Delay in repatriation

Capacity/system barriers	Delay in repatriation
Referred from other centre	Capacity/system barriers
Capacity/system barriers	Rehabilitation or discharge plannign delay
